# Building a predictive model for pregnancy outcomes in ART patients based on AMH, FORT, HCG day EMT, and clinical characteristics

**DOI:** 10.3389/fmed.2025.1699477

**Published:** 2025-11-27

**Authors:** Lei Zhang, Xiaofang Liu, Jiaojiao Bai, Yushan Wang

**Affiliations:** Department of Obstetrics and Reproductive Medicine, Chinese People’s Liberation Army Air Force Specialty Medical Center, Beijing, China

**Keywords:** assisted reproductive technology, pregnancy outcomes, human chorionic gonadotropin, endometrial thickness, serum anti-Müllerian hormone, follicle output rate

## Abstract

**Objective:**

To establish a predictive model for pregnancy outcomes in patients undergoing assisted reproductive technology (ART) treatment using human chorionic gonadotropin (HCG) day endometrial thickness (EMT), follicle output rate (FORT), serum anti-Müllerian hormone (AMH), and clinical characteristics.

**Methods:**

A retrospective study was conducted on 200 patients who underwent ART treatment at our hospital from August 2019 to August 2024. All patient data were obtained from the electronic medical record system, and patients were divided into a clinical pregnancy group and a non-pregnancy group based on whether they achieved pregnancy. Clinical-related data were collected and compared. Two machine learning methods, Lasso regression and Extreme Gradient Boosting (XGBoost), were used for “overlap coverage” screening of risk factors. The XGBoost model was implemented using the “xgboost” package in R, with the following hyperparameters: max_depth = 6, learning_rate = 0.3, n_estimators = 100, objective = “binary:logistic.” These parameters were selected based on common practices for binary classification tasks and were not extensively tuned due to the sample size limitations. Multivariate logistic analysis was performed to identify risk factors for pregnancy outcomes in ART patients. A regression model was established using R software for prediction and validation.

**Results:**

A total of 200 ART patients were included in this study. According to medical record system records, 109 patients did not achieve pregnancy, accounting for 54.50%, and were included in the non-pregnancy group. 91 patients achieved clinical pregnancy, accounting for 45.50%, and were included in the clinical pregnancy group. There were significant statistical differences between the non-pregnant group and the clinically pregnant group in terms of age, body mass index, number of oocytes retrieved, HCG day EMT, AMH, and FORT (*p* < 0.05); Using LASSO regression and XGBoost machine learning, different risk factors were identified. Through the “overlap coverage” method, six common risk factors for pregnancy outcomes in ART patients were identified and included in the logistic regression model. The results indicated that age and body mass index were both risk factors influencing pregnancy outcomes in ART patients (OR = 1.196, 1.777, *p* < 0.05); the number of retrieved oocytes, HCG day EMT, AMH, and FORT were protective factors influencing pregnancy outcomes in ART patients (OR = 0.366, 0.382, 0.182, 0.862, *p* < 0.05); Based on the results of the logistic regression analysis, a risk prediction model for pregnancy outcomes in ART patients was constructed using a nomogram. The ROC curve showed an AUC value of 0.911, with a 95% confidence interval (CI) of 0.871 to 0.951. The model was validated using the Bootstrap method with 1,000 repeated samples, yielding a Cox-Snell R^2^ of 0.629, Nagelkerke R^2^ was 0.471, Brier Score was 0.117, model fit *p*-value was 0.240, and the statistic was 10.369. This indicates that the model’s predictive results align well with the actual occurrence of pregnancy outcomes in patients, with the clinical decision curve generally exceeding the two extreme curves. This suggests that the factors included in the nomogram provide a high net benefit for predicting pregnancy outcomes in ART-treated patients.

**Conclusion:**

There are numerous factors influencing pregnancy outcomes in ART patients, primarily including age, body mass index, number of oocytes retrieved, HCG day EMT, AMH, and FORT. The risk prediction nomogram model constructed based on these factors has certain predictive value for pregnancy outcomes in ART patients. Clinicians should identify high-risk populations early and optimize treatment plans accordingly.

## 1 Introduction

Assisted reproductive technology (ART) has undergone more than 40 years of development, with over 2 million cycles performed globally each year. However, the clinical pregnancy rate ranges from 40 to 50%, and the live birth rate is even lower, highlighting the urgent need to optimize predictive models for pregnancy outcomes (1, 2). Bianchi C et al. (3) noted that the current bottlenecks affecting ART success rates primarily stem from two core factors: embryo quality and endometrial receptivity. The former is closely related to ovarian responsiveness and oocyte developmental potential, while the latter involves endometrial receptivity. Together, these two factors form the “dual-factor balance” model of embryo implantation. With the rapid advancement of embryo culture and genetic screening technologies, embryo selection has achieved precision, but endometrial receptivity assessment remains heavily reliant on subjective indicators such as ultrasound morphology, lacking standardized biological markers. Furthermore, Gao R et al. (4) noted that there is a dynamic interaction between ovarian reserve function and endometrial receptivity. This bidirectional regulatory mechanism limits the predictive value of single indicators, necessitating the establishment of a multidimensional predictive system that integrates endometrial status and ovarian function.

Previous studies have indicated that endometrial receptivity refers to the specialized functional state of the endometrium that allows embryo adhesion, invasion, and induces endometrial stromal decidualization. Its assessment has historically been a challenge in the field of assisted reproductive technology (ART). Human chorionic gonadotropin (HCG)-day endometrial thickness (EMT) has become the most widely used clinical evaluation indicator due to its non-invasive and quantifiable nature (5, 6). Related clinical studies have pointed out that an EMT of 7–12 mm at the time of ovulation or embryo transfer is ideal, as the endometrium is rich in glands and blood flow at this time, providing a suitable microenvironment for embryo implantation (7, 8). Grynberg M et al. (9) noted that ovarian function indicators such as serum anti-Müllerian hormone (AMH) and follicle output rate (FORT) can effectively quantify ovarian reserve, reflecting the ovarian response to exogenous stimulation and the quality of the follicular microenvironment. AMH is secreted by granulosa cells in preantral and small antral follicles, and its serum concentration directly reflects the size of the primordial follicle pool, making it the most sensitive indicator of ovarian reserve function. The core value of FORT lies in quantifying the sensitivity of the ovaries to gonadotropin stimulation. Unlike AMH, which only reflects static reserve, FORT dynamically captures the conversion efficiency of follicles from recruitment to maturation (10, 11). These indicators cover key stages from ovarian stimulation to embryo implantation in ART cycles. Their combined assessment may overcome the limitations of traditional single-indicator predictions, offering new perspectives for personalized treatment. It is worth noting that with the significant increase in the proportion of frozen–thawed embryo transfer (FET) cycles, non-physiological endometrial preparation protocols further alter the endometrial-embryo synchronization mechanism, making predictive models more complex. Existing models are often limited to a single dimension.

Recent studies have increasingly utilized machine learning (ML) for predicting ART outcomes. For instance, Liu et al. developed an XGBoost model incorporating age, BMI, and ovarian reserve markers to predict live birth, achieving an AUC of 0.89 (12). Similarly, Zhang et al. used a random forest classifier with endometrial and embryological features, reporting improved prediction accuracy over traditional logistic regression (13). Chen et al. applied a deep neural network to predict implantation success, highlighting the value of integrating multi-omics data (14). Wang et al. compared several ML algorithms and found ensemble methods like XGBoost to be superior in handling nonlinear relationships in ART data (15). Finally, Li et al. demonstrated the utility of LASSO for feature selection in a large multicenter ART cohort, reinforcing the importance of variable reduction in high-dimensional clinical settings (16). These studies underscore the potential of ML in enhancing ART outcome prediction. However, many existing models focus on a limited set of predictors or single dimensions (e.g., embryological or endometrial factors alone). In contrast, our study integrates both ovarian function indicators (AMH, FORT) and endometrial receptivity marker (HCG day EMT) alongside key clinical characteristics (age, BMI, number of oocytes retrieved) using a hybrid machine learning approach (LASSO and XGBoost) for feature selection. This comprehensive variable selection strategy, followed by logistic regression modeling, allowed us to develop a nomogram with an AUC of 0.911, which compares favorably to the previously reported models (e.g., Liu et al., AUC = 0.89; Zhang et al., improved accuracy over logistic regression). Our model’s performance, particularly its high discriminative ability and net benefit as shown by the decision curve analysis, suggests that the integration of multifaceted factors may offer superior predictive value for ART pregnancy outcomes compared to models relying on narrower sets of predictors. Based on this, this study selected patients undergoing ART treatment at our hospital for analysis, systematically examining the predictive value of endometrial thickness and ovarian function indicators on pregnancy outcomes on the day of hCG administration. By developing an individualized treatment decision support system using machine learning algorithms, this research aims to advance ART treatment toward precision medicine, ultimately improving pregnancy outcomes.

## 2 Materials and methods

### 2.1 Declaration of ethical standards

This study was approved by the review committee and ethics committee of this institution. Given that this study is a retrospective study and only de-identified patient data was used, informed consent was not required as there was no risk or adverse effect to patients. This exemption complies with regulations and ethical guidelines related to retrospective studies.

### 2.2 Study design

This retrospective study included 200 patients who received ART treatment at our hospital between August 2019 and August 2024. All patient data were obtained from the electronic medical record system and divided into clinical pregnancy and non-pregnancy groups based on pregnancy status.

### 2.3 Inclusion criteria

Inclusion Criteria: (1) Age > 22 years; (2) Complete clinical records; (3) All meet the clinical indications specified in the “Guidelines for Human Assisted Reproductive Technology” (17); (4) No concomitant severe neurological disorders or cognitive impairments.

Exclusion Criteria: (1) Presence of uterine malformations; (2) History of ovarian surgery; (3) Presence of organ dysfunction (e.g., cardiac, hepatic, renal); (4) Presence of infectious diseases.

### 2.4 Treatment methods

All patients first undergo ovulation induction therapy, which includes the following protocols: long-acting gonadotropin-releasing hormone (GnRH) protocol, modified long-acting GnRH protocol, GnRH antagonist protocol, and other superovulation protocols. During the superovulation period, clinicians assess follicle development based on vaginal ultrasound and serum hormone levels. When the dominant follicle diameter reaches the standard (typically ≥3 follicles ≥18 mm in diameter), human chorionic gonadotropin (HCG) is administered to induce final follicle maturation. Oocyte retrieval is performed approximately 36 h after HCG injection under vaginal ultrasound guidance. Within 4–6 h after egg retrieval, conventional *in vitro* fertilization (IVF) or intracytoplasmic sperm injection (ICSI) is selected based on patient indications (e.g., semen parameters). Fertilization is observed 16–20 h post-fertilization. After 72 h, embryo quality is assessed, and 1–2 embryos are selected for transfer. Embryo transfer: ① Fresh embryo transfer: For patients who did not experience moderate to severe ovarian hyperstimulation syndrome (OHSS), significant ascites, or other conditions unsuitable for fresh cycle transfer (such as premature progesterone elevation) during the ovulation induction and egg retrieval stages, fresh transfer of cleavage-stage embryos is performed on day 3 post-retrieval. Starting from the day of egg retrieval or embryo transfer, all patients undergoing embryo transfer receive luteal support (intramuscular progesterone or vaginal progesterone preparations) until serum *β*-HCG levels are measured 2 weeks after embryo transfer. ② Frozen–thawed embryo transfer: For patients who develop moderate to severe OHSS risk or symptoms, significant ascites, premature elevation of progesterone, or other clinical conditions deemed unsuitable for fresh transfer during the ovulation induction or egg retrieval phase, all embryos are cryopreserved via vitrification. After the patient’s condition improves, endometrial preparation is conducted via either natural cycle ovulation monitoring or hormone replacement therapy based on the patient’s condition. When the endometrial thickness reaches the ideal state, the cryopreserved embryos are thawed and transferred. In a natural cycle, luteal support is initiated post-ovulation or on the day of embryo transfer, following the same protocol as in a fresh cycle. In a hormone replacement cycle, estradiol is administered from the start of estrogen supplementation to establish the endometrium. Once the endometrium meets the criteria, progesterone is added to facilitate endometrial transformation, and embryo transfer is performed on the scheduled transfer day. Luteal support continues according to the same protocol as in a fresh cycle.

### 2.5 Grouping method

Serum *β*-HCG levels are measured 14 days after embryo transfer to determine biochemical pregnancy (serum β-HCG ≥ 10 mIU/mL is considered positive). A transvaginal ultrasound is performed 28 days after embryo transfer to detect an intrauterine gestational sac and primitive heartbeat to confirm clinical pregnancy. Otherwise, it is considered a non-pregnancy.

### 2.6 General data collection

General patient data were retrospectively collected through the electronic medical record system, primarily including age, body mass index, family history (yes/no), smoking history (yes/no, defined as daily smoking volume > 1 cigarette, with a continuous duration > 1 year or cessation period < 1 year), alcohol consumption history (defined as daily alcohol intake > 1 drink unit, duration > 1 year, or abstinence < 1 year, where 1 drink unit is equivalent to 45 mL of spirits/360 mL of beer/120 mL of wine) (yes/no), Hypertension [meeting the diagnostic criteria in The Japanese Society of Hypertension Guidelines for Self-monitoring of Blood Pressure at Home (Second Edition) (18)] (Yes/No), Diabetes [meeting the diagnostic criteria in Application of the Chinese Expert Consensus on Diabetes Classification in clinical practice (19)] (Yes/No), Hyperlipidemia [meeting the diagnostic criteria in the “Report of the Japan Atherosclerosis Society (JAS) Guideline for Diagnosis and Treatment of Hyperlipidemia in Japanese Adults” (20)] (Yes/No), duration of infertility, total number of Gn days, initial Gn dose, total Gn dose, number of oocytes retrieved, number of fertilized oocytes, type of transferred embryos, number of cleaved embryos, number of transferred embryos.

### 2.7 HCG day EMT measurement

All patients were treated with individualized ovulation induction protocols. When three or more follicles reached a diameter of ≥18 mm, HCG was administered to promote final oocyte maturation. On the morning of the HCG injection day, EMT was measured using transvaginal ultrasound.

### 2.8 AMH and FORT testing

Five milliliters of fasting venous blood was drawn from the patient in the morning during the menstrual cycle 1 month prior to egg retrieval. A centrifuge (manufacturer: Beijing Shidai Beili Centrifuge Co., Ltd.) was used at 3000 r/min for 10 min to separate the patient’s serum. AMH was then tested using chemiluminescence and (Yahuilong Company).

### 2.9 Statistical analysis

This study utilized SPSS 26.0 and R 4.3.2 software for data statistical analysis and visualization. For metric data following a normal distribution, independent samples t-tests were performed, with results presented as (x̅ ± s). For non-normally distributed data, Mann–Whitney U tests were conducted, with results presented as [M (P25, P75)]. For comparisons of categorical variables, chi-square (χ^2^) tests were used, with results expressed as frequencies. The significance level was set at *p* < 0.05. The study employed two machine learning methods—LASSO regression and XGBoost—for variable selection. ASSO regression minimizes the following objective function: min_β_{1/2 N∑^N^_i = 1_(yi − β0 − ∑^p^_j = 1_x_ij_β_j_)^2^ + *λ*∑^p^_j = 1_∣βj∣}, where y_i_ is the outcome, x_ij_ are the predictors, β_j_ are the coefficients, and λ is the penalty parameter. XGBoost builds an ensemble of decision trees by iteratively adding trees that predict the residuals of previous trees. The objective function for step t is: L^(t)^ = ∑^n^_i = 1_l(y_i_,y^_i_^(t − 1)^ + f_t_(x_i_)) + *Ω*(f_t_) where Ω(f_t_) = *γ*T + 1/2λ∑^T^_j = 1_w^2^_j_, with T being the number of leaves, w_j_ the leaf weights, and γ, λ regularization parameters. The overlapping and overlapping important risk factors selected by the two machine learning methods were analyzed, and the selected variables were included in logistic regression analysis. A prediction model based on the rms package was developed, and internal validation was conducted using Bootstrap resampling (1,000 times). ROC curve analysis was used to calculate the AUC value to evaluate discriminative ability, calibration curves were plotted to assess predictive accuracy, and decision curves were applied to evaluate clinical utility.

## 3 Results

### 3.1 Comparison of clinical data between the non-pregnant group and the clinically pregnant group

A total of 200 patients undergoing ART treatment were included in this study. According to medical record system records, 109 patients did not become pregnant, accounting for 54.50%, and were included in the non-pregnant group. Ninety-one patients achieved clinical pregnancy, accounting for 45.50%, and were included in the clinically pregnant group. There were significant statistical differences between the non-pregnant group and the clinically pregnant group in terms of age, body mass index, number of oocytes retrieved, HCG day EMT, AMH, and FORT (*p* < 0.05). However, there were no significant statistical differences in terms of family history and other variables (*p* > 0.05), as shown in Table 1.

**Table 1:** Comparison of clinical data between the non-pregnant group and the clinically pregnant group.

Indicator		Non-pregnant group (*n* = 109)	Clinical pregnancy group (*n* = 91)	*χ*^2^/*t/Z*	*p*
Age (years)		34.99 ± 4.01	32.70 ± 4.07	3.994	0.001
Body Mass Index (kg/m^2^)		23.09 ± 1.21	22.44 ± 1.17	3.840	<0.001
Family history (n)	have	4	2	0.369	0.543
	no	105	89		
History of smoking (n)	have	19	11	1.831	0.176
	no	90	90		
History of alcohol consumption (n)	have	24	13	1.967	0.161
	no	85	78		
hypertension (n)	have	3	2	0.063	0.802
	no	106	89		
diabetes (n)	have	2	1	0.182	0.670
	no	107	90		
Hyperlipidemia (n)	have	4	2	0.369	0.543
	no	105	89		
Years of infertility (years)		4.48 ± 2.20	4.39 ± 2.34	0.280	0.780
Total days of Gn (d)		10.74 ± 1.76	10.94 ± 1.88	0.776	0.439
Gn initiation quantity (U)		251.21 ± 55.82	249.88 ± 55.02	0.169	0.866
Total Gn (U)		2755.34 ± 837.99	2794.83 ± 841.79	0.327	0.744
Number of retrieved oocytes (units)		8.37 ± 1.09	9.34 ± 1.34	5.645	<0.001
Fertilization number (units)		8.02 ± 4.93	8.71 ± 4.15	1.058	0.291
Type of transplanted embryo (n)	Cleavage-stage embryo	89	78	0.594	0.441
	Blastocyst	20	13		
Number of cleavages (units)		7.88 ± 3.67	8.15 ± 3.91	0.503	0.616
The number of transplanted embryos (units)		1.81 ± 0.40	1.89 ± 0.31	1.557	0.121
HCG Day EMT (mm)		9.85 ± 1.01	10.78 ± 1.15	6.087	<0.001
AMH(ng/mL)		3.23 ± 0.41	3.51 ± 0.45	4.600	<0.001
FORT(%)		82.36 ± 5.53	86.92 ± 6.28	5.459	<0.001

### 3.2 Analysis of important variables affecting pregnancy outcomes in ART patients

**Figure 1:**
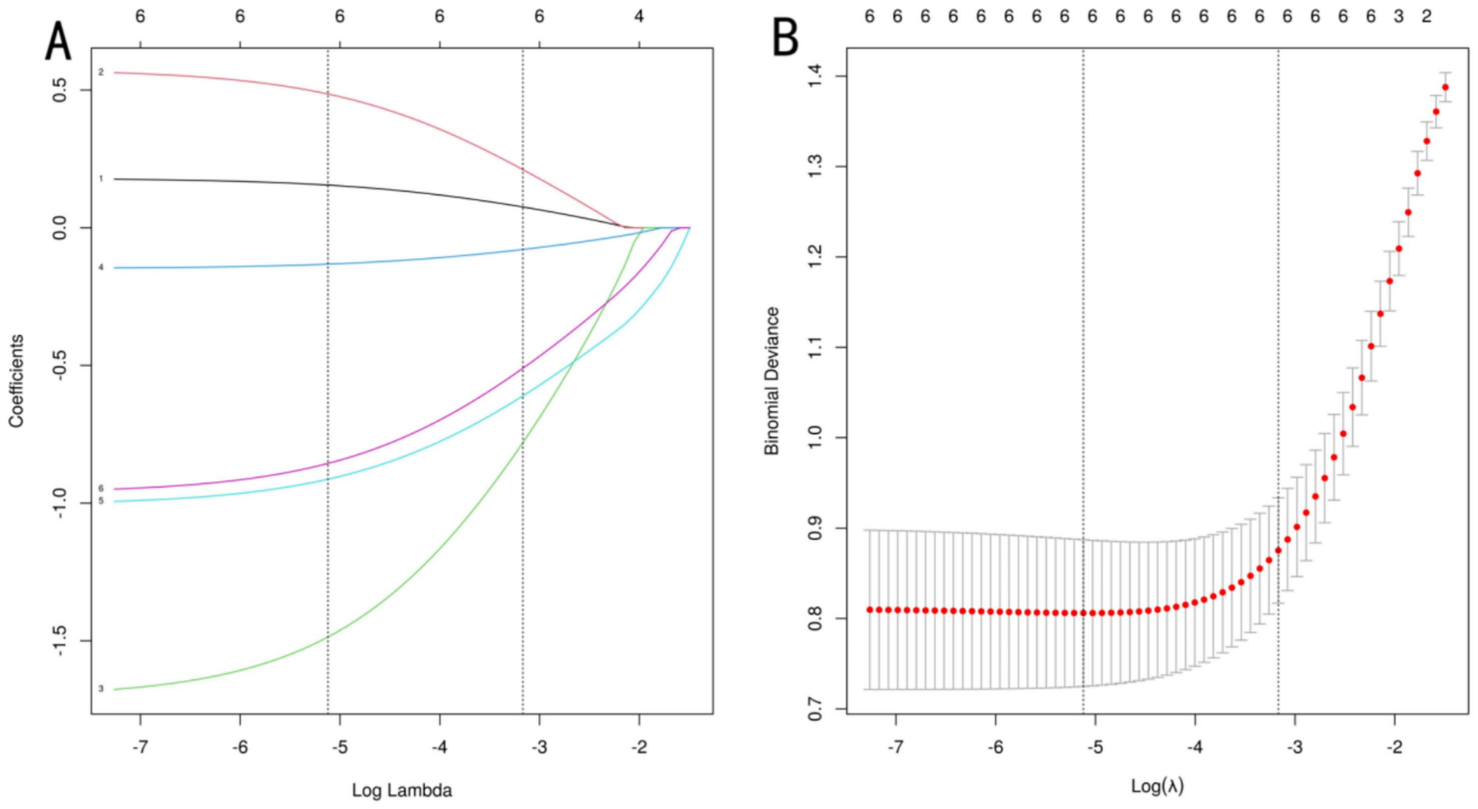
LASSO regression analysis. **(A)** uses a dual horizontal axis design, with the lower axis displaying the logarithmic value of log(λ) and the upper axis displaying the number of retained variables. The vertical axis presents the standardized coefficients of each feature, with their change trajectories intuitively displayed through colored curves. **(B)** shows the mean squared error on the vertical axis. The red vertical line indicates the optimal variable subset corresponding to the minimum error value λ.min, which represents the number of independent variables in the model when the mean squared error is minimized. The black vertical line λ.lse represents the simplified model selection when the error increases by one standard error.

To optimize model performance and address multicollinearity issues, the study used LASSO regression to perform feature selection on the initially screened differential variables (Figure 1A), compressing the regression coefficients of redundant variables to 0. To validate the reliability of the LASSOlogit results, cvlassologit cross-validation was used to determine the optimal *λ* (Figure 1B). Ultimately, the best fitting results were achieved when λ was 0.006 for minimum mean square error and 0.042 for minimum distance standard error, with six variables included: age, body mass index, number of oocytes retrieved, HCG day EMT, AMH, and FORT. According to XGBoost analysis, the proportions of age, body mass index, number of oocytes retrieved, HCG day EMT, AMH, and FORT were 12, 9, 24, 30, 12, and 13%, respectively, demonstrating clinical interpretability. By applying an “overlap coverage” analysis to the two machine learning models’ parallel evaluations of important variables, six meaningful factors were jointly identified: age, body mass index, number of oocytes retrieved, HCG day EMT, AMH, and FORT.

**Figure 2:**
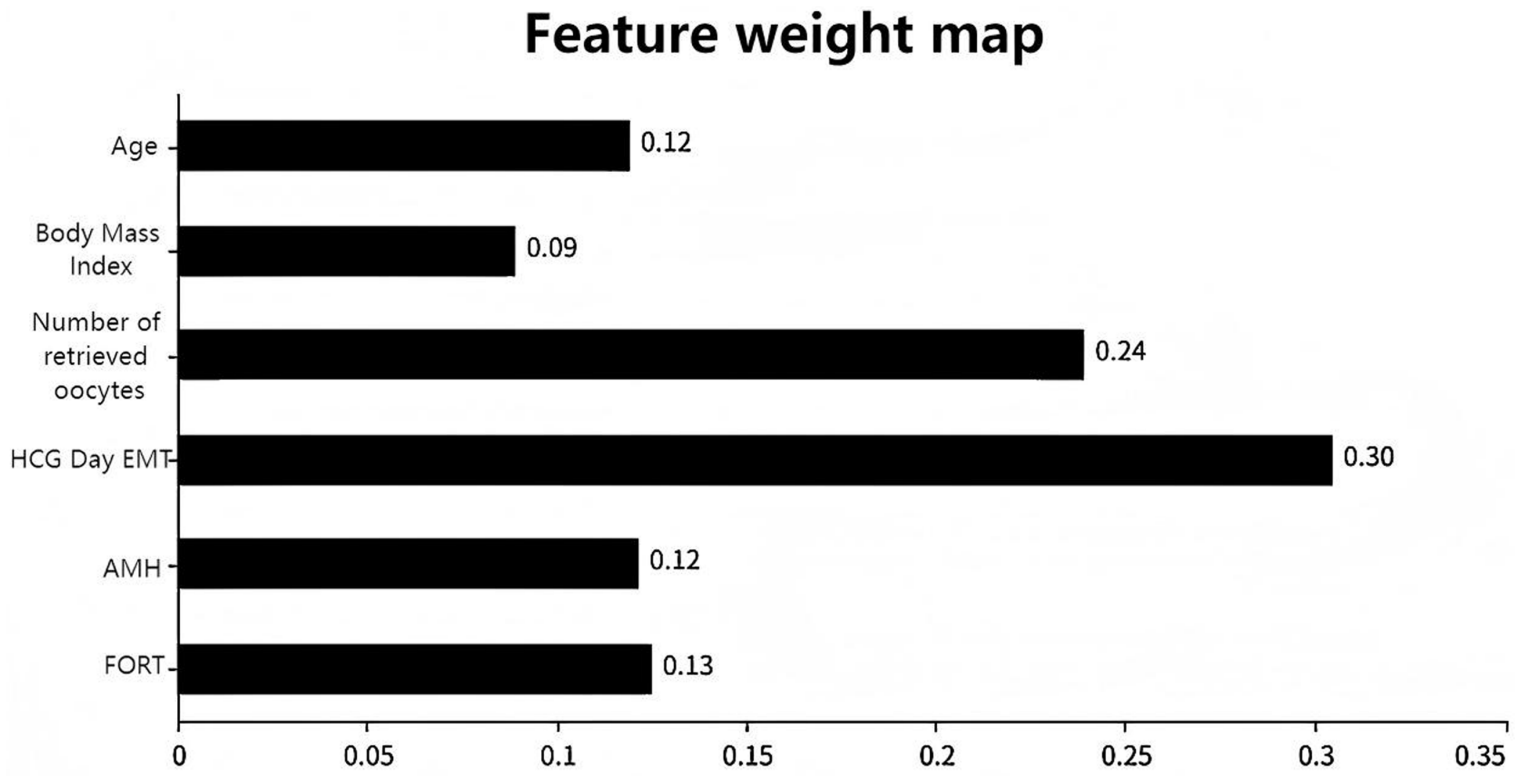
XGBoost feature importance plot.

Figure 1A uses a dual horizontal axis design, with the lower axis displaying the logarithmic value of log(*λ*) and the upper axis displaying the number of retained variables. The vertical axis presents the standardized coefficients of each feature, with their change trajectories intuitively displayed through colored curves. Figure 1B shows the mean squared error on the vertical axis. The red vertical line indicates the optimal variable subset corresponding to the minimum error value λ.min, which represents the number of independent variables in the model when the mean squared error is minimized. The black vertical line λ.lse represents the simplified model selection when the error increases by one standard error. Figure 2 shows the importance scores of the variables identified by the XGBoost model, with AMH having the highest score and age having the lowest.

### 3.3 Logistic regression analysis of factors influencing pregnancy outcomes in patients undergoing ART treatment

Variables selected using LASSO regression and XGBoost algorithms were included in the logistic analysis. The results indicated that age and body mass index were both risk factors influencing pregnancy outcomes in patients undergoing ART treatment (OR = 1.196, 1.777, *p* < 0.05), while the number of oocytes retrieved, HCG day EMT, AMH, and FORT were protective factors influencing pregnancy outcomes in patients undergoing ART treatment (OR = 0.366, 0.382, 0.182, 0.862, *p* < 0.05), as shown in Table 2.

**Table 2:** Logistic regression analysis on the pregnancy outcomes of patients treated with ART.

Project	Coefficient of regression	standard error	*z* value	Wald χ^2^	*p* value	OR value	OR value 95% CI
Age	0.179	0.055	3.238	10.486	0.001	1.196	1.073–1.334
Body Mass Index	0.575	0.197	2.926	8.560	0.003	1.777	1.209–2.612
Number of retrieved oocytes	−1.006	0.238	−4.227	17.868	0.000	0.366	0.229–0.583
HCG Day EMT	−0.963	0.218	−4.416	19.504	0.000	0.382	0.249–0.585
AMH	−1.706	0.537	−3.176	10.086	0.001	0.182	0.063–0.520
FORT	−0.148	0.040	−3.715	13.798	0.000	0.862	0.798–0.932

### 3.4 Development of a risk-stratification model for predicting pregnancy outcomes in patients undergoing ART treatment

**Figure 3:**
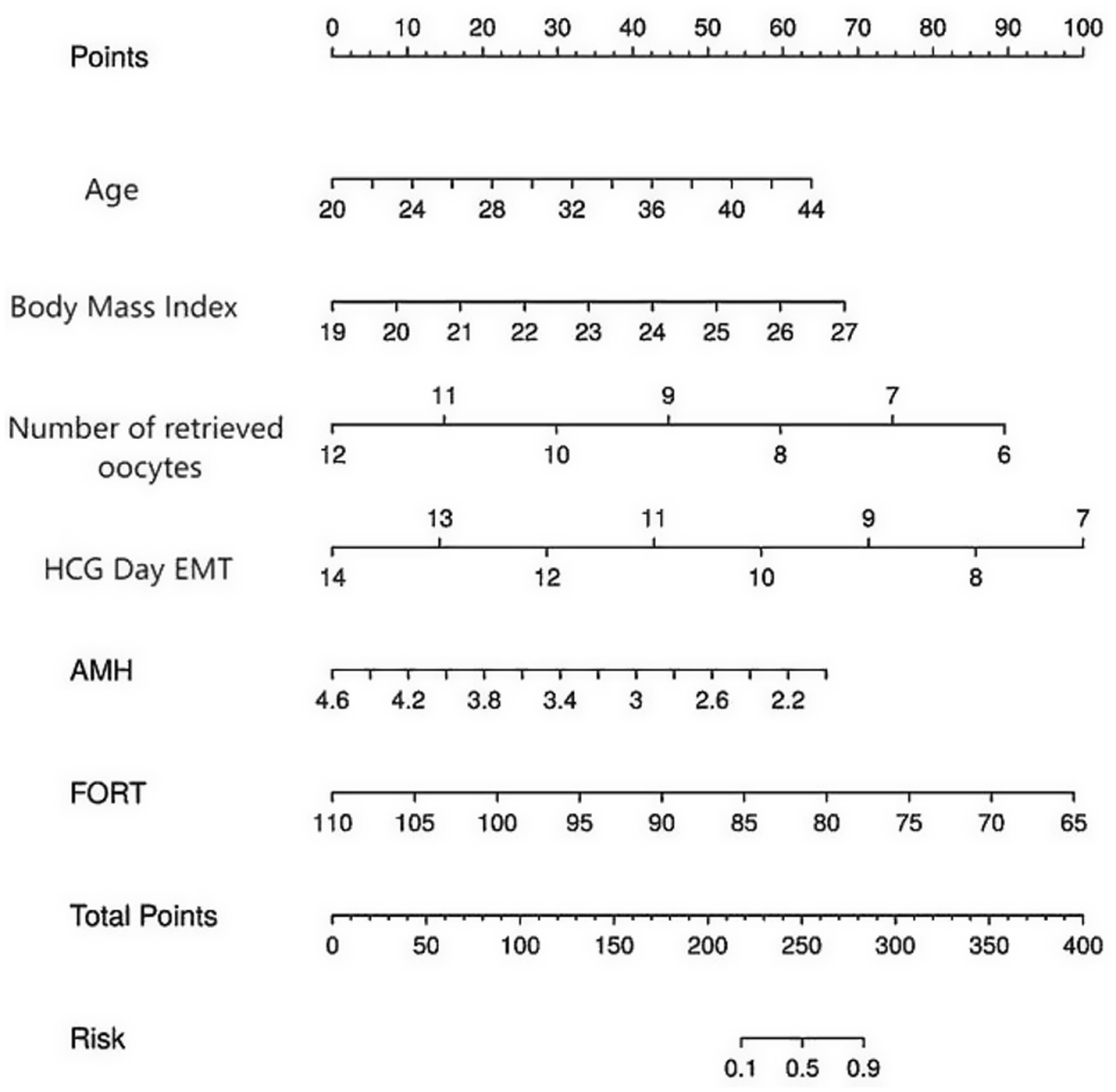
A risk prediction nomogram for pregnancy outcomes in patients treated with ART. The nomogram is used by summing the points assigned to each variable (Age, BMI, Number of Retrieved Oocytes, HCG Day EMT, AMH, FORT) and projecting the total points to the “Total Points” axis. The corresponding value on the “Risk of Non-pregnancy” axis indicates the predicted probability of non-pregnancy.

Based on the results of the statistical analysis, the following regression model was established: Logit(P) = −18.290 + 0.179 × age + 0.575 × body mass index—1.006 × number of oocytes retrieved—0.963 × HCG day EMT—1.706 × AMH - 0.148 × FORT. Based on the six independent predictive factors identified through screening, a visual scoring system was developed. Each risk factor corresponds to an independent scale axis, with the scale length intuitively reflecting the weight of that factor’s contribution to the prognosis. The total score is obtained by summing the scores corresponding to each variable, and the individualized risk prediction value is mapped onto the probability scale, enabling a quantitative assessment of pregnancy outcomes in ART patients, as shown in Figure 3.

### 3.5 Model performance evaluation

#### 3.5.1 ROC curve

**Figure 4:**
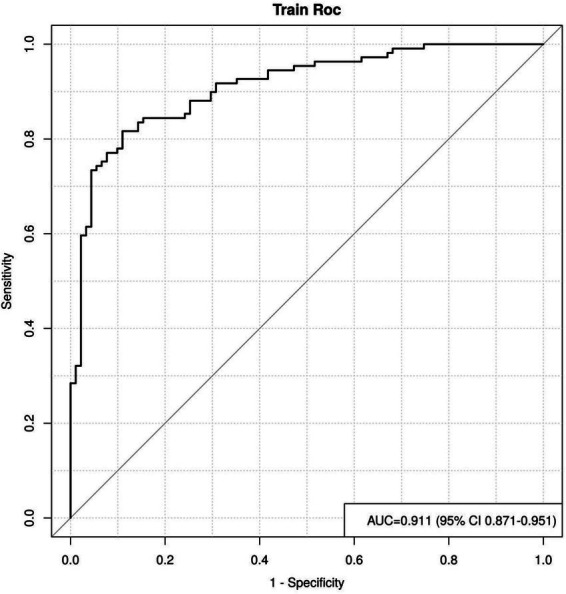
The area under the ROC curve of the nomogram model.

By plotting the ROC curve, we can see that the AUC value is 0.911, with a 95% CI of 0.871 to 0.951, indicating that the reclassified curve model has good discriminatory power, as shown in Figure 4.

#### 3.5.2 Calibration curve and decision curve

**Figure 5:**
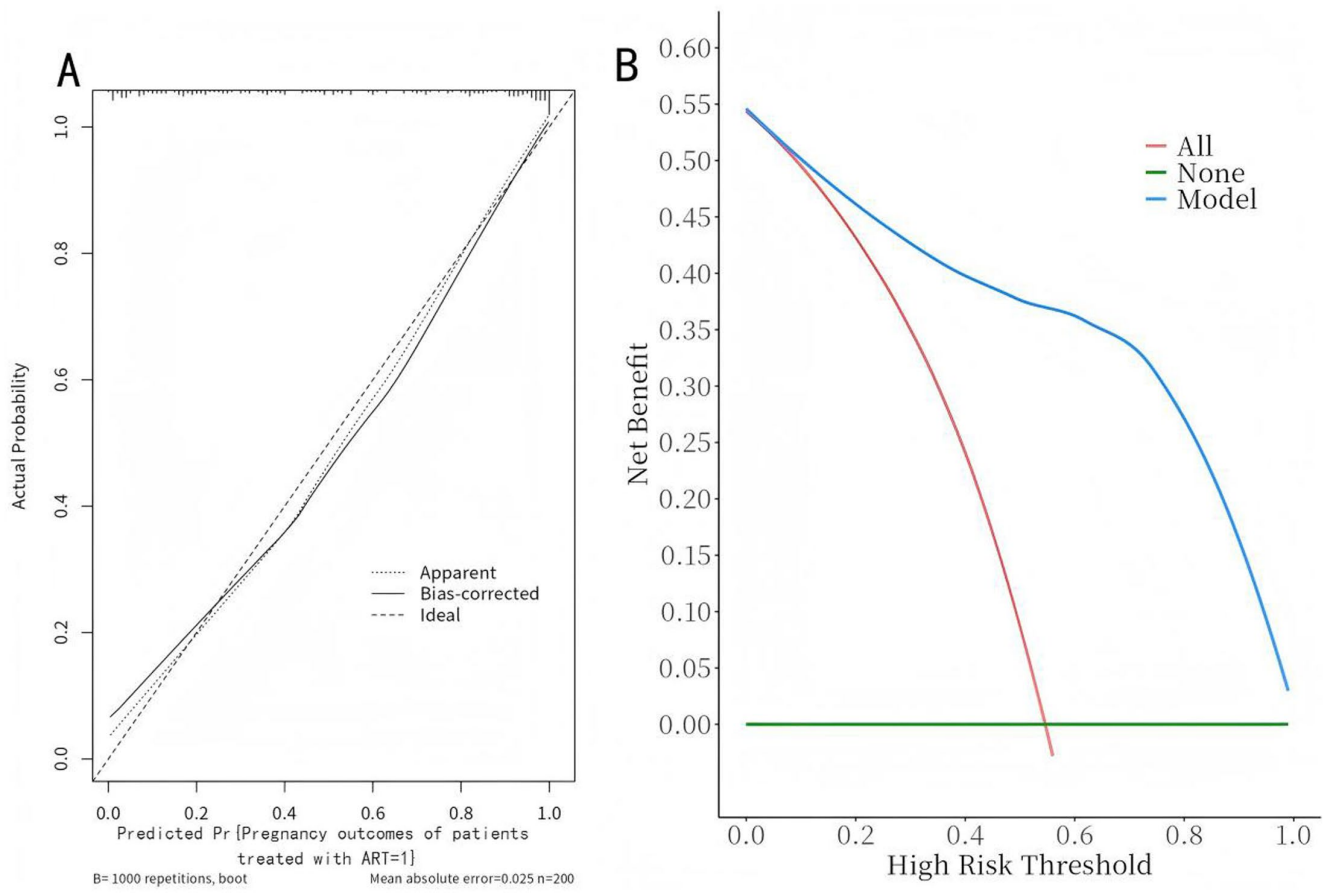
Calibration curves and decision curves of the nomogram model. **(A)** Calibration curve: The x-axis represents the predicted probability of non-pregnancy, and the y-axis represents the actual observed frequency. The dashed line is the reference line of perfect prediction. The solid line indicates the performance of the nomogram. Pregnancy Outcomes in ART-Treated Patients (=1). **(B)** Decision curve: The x-axis represents the threshold probability, and the y-axis represents the net benefit. The solid black line represents the nomogram, the gray line “None” assumes no patients experience the event, and the black line “All” assumes all patients experience the event.

The model was validated using the Bootstrap method with 1,000 repeated samples. The Cox-Snell R^2^ was 0.629, the Nagelkerke R^2^ was 0.471, the Brier Score was 0.117, and the model fit *p*-value was 0.240. the statistic is 10.369, indicating that the column plot model exhibits good calibration, as shown in Figure 5A; the DCA curve is higher than the two extreme curves, indicating that the net predictive gain of the relevant factors in the column plot is high, as shown in Figure 5B.

## 4 Discussion

ART is currently an important method for treating infertility. During treatment, patients generally use the same ovulation-inducing drugs, and techniques such as gamete handling and embryo culture are relatively mature (21, 22). Graham ME et al. (23) noted that ART is a medical technique that assists infertile couples in achieving pregnancy, encompassing two main categories: artificial insemination and *in vitro* fertilization-embryo transfer (IVF-ET) and its derivative techniques. On one hand, exogenous gonadotropins are used to induce synchronized development of multiple follicles, combined with GnRH analogs to precisely trigger ovulation at the optimal time, thereby obtaining an adequate number of high-quality oocytes; on the other hand, after oocytes are retrieved, fertilization is completed in vitro, directly addressing severe male infertility issues. Embryos are cultured to the cleavage stage or blastocyst stage, with precise regulation of gamete development, fertilization environment, embryo selection, and endometrial status, forming a multi-step synergistic effect. This ultimately overcomes the physiological limitations of natural conception, reduces miscarriage rates, and improves live birth rates (24). However, some patients still experience poor pregnancy outcomes due to the combined effects of multiple factors, which severely impact both physical and mental health. Therefore, actively analyzing risk factors influencing pregnancy outcomes in ART patients and constructing predictive models is of significant clinical importance for implementing corresponding preventive and therapeutic measures.

HCG day EMT refers to the endometrial thickness measured on the day of HCG injection in assisted reproductive technology, which is crucial for assessing endometrial receptivity, embryo implantation success rates, and pregnancy outcomes; FORT refers to the ratio of the number of mature follicles with a diameter ≥14 mm in both ovaries on the day of HCG injection to the number of antral follicles with a diameter of 2–8 mm before ovulation induction, which is an important indicator for assessing ovarian responsiveness; AMH is secreted by preantral follicles and small antral follicles in the ovaries, and its levels are directly related to the number of remaining follicles in the ovaries. It is used to assess ovarian reserve function, reflecting the quantity and quality of remaining oocytes in the ovaries, and is closely associated with the efficacy of reproductive treatment (25–27). Based on the data from this study, significant differences were observed between the non-pregnant group and the clinically pregnant group in terms of EMT, AMH, and FORT on the day of HCG administration (*p* < 0.05). Logistic regression analysis indicated that AMH, EMT on the day of HCG administration, and FORT are all risk factors influencing pregnancy outcomes in patients undergoing ART (p < 0.05). Previous studies have indicated that synchronized endometrial development is essential for embryo implantation. When EMT > 10 mm, estrogenization is optimal, facilitating embryo implantation; when EMT is between 7 mm and 10 mm, the pregnancy rate increases with increasing EMT (28). Martins RS et al. (29) reported that insufficient EMT on the day of HCG administration is an important risk factor for pregnancy failure in ART cycles, primarily involving impaired endometrial receptivity, impaired blood flow perfusion, and abnormal molecular microenvironment. On one hand, a decrease in EMT on the HCG day is often accompanied by increased uterine spiral artery blood flow resistance, delayed glandular epithelial development, and reduced vascular endothelial growth factor expression, leading to inadequate endometrial blood supply, reduced oxygen and nutrient delivery, and impaired normal endometrial proliferation and decidualization. When the endometrium is too thin, receptor density is insufficient, weakening the role of HCG in promoting endometrial thickening and establishing receptivity; On the other hand, decreased EMT on the HCG day indicates endometrial thinning, and inadequate decidualization may fail to support early placental formation, leading to endometrial receptivity defects, ectopic implantation of the embryo, and an increased risk of clinical non-pregnancy. Under normal physiological conditions, AMH has a stimulatory effect on follicle development and can also prevent excessive follicle depletion to some extent, thereby enhancing ovarian reserve function. Referring to the study by Peigné M et al. (27), estrogen and progesterone imbalance is an important cause of irregular changes in the endometrium. AMH is a glycoprotein differentiation factor primarily produced by granulosa cells of primordial follicles, which can enter the systemic circulation via follicular fluid. Serum AMH levels fundamentally reflect ovarian antral follicle reserve. Abnormal serum AMH levels may impair oocyte maturation, thereby reducing egg and embryo quality, leading to poor embryo quality and adverse pregnancy outcomes. Previous studies have indicated that FORT is a novel assessment indicator applied in clinical reproductive systems in recent years, first proposed by Genro et al. in 2011. It objectively reflects the developmental potential of follicles within the ovarian follicle, independent of the original number of follicles in the ovarian follicle, and is instead correlated with the overall health status of follicles (30). FORT assessment is an important indicator for predicting the extent of follicle response to medication. Its expression is not influenced by factors such as age, exhibiting uniqueness and high stability. It quantitatively assesses the follicular potential of the ovaries in patients with relatively regular menstrual cycles. As FORT values increase, the number of retrieved oocytes and high-quality embryos also increases, playing a positive role in achieving favorable assisted reproductive outcomes.

The data from this study revealed significant statistical differences in age, body mass index, and number of oocytes retrieved between the non-pregnant group and the clinically pregnant group (*p* < 0.05). Logistic regression analysis indicated that age, body mass index, and number of oocytes retrieved are all risk factors influencing pregnancy outcomes in patients undergoing ART treatment (*p* < 0.05). Previous studies have indicated that overweight and obesity are closely associated with clinical symptoms, hormonal abnormalities, and lipid metabolism abnormalities in infertile patients (31). Sex hormones in the human body are primarily metabolized through adipose tissue. Abnormal increases in adipose tissue can lead to abnormalities in drug metabolism kinetics, resulting in reduced levels of follicle-stimulating hormone (FSH) and subsequently affecting follicle quality (32). Referring to the study by Li YL et al. (33), it is known that in obese women, more leptin in follicles originates from adipose tissue. Therefore, leptin significantly inhibits the stimulatory effect of follicle-stimulating hormone on steroid synthesis in granulosa cells, leading to reduced ovarian responsiveness and increased risk of clinical non-pregnancy. In this study, low oocyte retrieval numbers were identified as a core risk factor for treatment failure in ART. The mechanism may involve a dual decline in both oocyte quantity and quality due to reduced ovarian reserve function and low ovarian responsiveness. Insufficient oocyte retrieval directly limits the acquisition of euploid embryos, while egg quality deteriorates simultaneously, with increased granulosa cell apoptosis and impaired cumulus cell function, affecting oocyte maturation and subsequent embryonic developmental potential. This further reduces the number of available eggs, negatively impacts endometrial receptivity, and impedes embryo implantation, leading to ART treatment failure through a combination of egg quantity deficiency, reduced embryonic developmental potential, and endometrial receptivity imbalance (34). Female fertility begins to decline after the age of 35 and shows a significant decline after the age of 37, with an irreversible decline as age increases. The number of oocytes decreases, and egg aging accelerates, manifested as mitochondrial dysfunction and metabolic disorders, leading to a significant reduction in fertilization rates and embryonic development potential, which is an important factor contributing to poor pregnancy outcomes. Additionally, studies have shown that aging leads to reduced estrogen receptor expression, decreased endometrial blood flow perfusion, and inadequate glandular development in women. Even with exogenous hormone supplementation, adequate proliferation is difficult to achieve, resulting in reduced endometrial thickness, which impedes embryo adhesion and placental formation. Systemic functional decline is exacerbated, and through the synergistic effects of germ cell aging, accumulated genetic abnormalities, and systemic metabolic imbalance, the risk of ART-related pregnancy failure increases (35).

In this study, a nomogram prediction model was constructed by combining various factors, transforming complex multivariate regression equations into graphical representations, thereby making abstract data outcomes visualizable and readable. Different predictive variables were used to assess the approximate probability of adverse pregnancy outcomes in patients. The AUC value of the ROC curve was 0.911, indicating that the model has excellent discriminative ability and can accurately distinguish between clinically pregnant and non-pregnant groups. The 95% CI (0.871–0.951) also indicates that this result is highly reliable. This performance is comparable to or even better than some recent ML-based ART prediction models, such as the XGBoost model by Liu et al. (AUC = 0.89) (12) and the random forest model by Zhang et al. (13), and outperforms traditional logistic regression models commonly used in clinical practice. The calibration curve shows that the model’s predictive results are well aligned with the actual occurrence of adverse pregnancy outcomes. This means that the consistency between the model’s predictive results and the actual observed outcomes is high, further enhancing the model’s credibility. The clinical decision curve is generally higher than the two extreme curves, indicating that the factors included in the nomogram have a high net benefit in predicting adverse pregnancy outcomes, providing stronger support for clinical decision-making. The integration of both ovarian and endometrial factors, along with core clinical characteristics, likely contributes to this robust performance, addressing the limitation of single-dimensional models highlighted in previous studies (3, 4). Limitations of this study: (1) Due to the limitations of single-center data collection, the small sample size reduces statistical power, potentially leading to a lack of representativeness in patient analysis; (2) Although the constructed nomogram model was validated using methods such as ROC curves, calibration curves, and decision curves, the validation was primarily based on internal validation and lacked corresponding external validation; (3) Long-term outcomes were not included, as this study primarily focused on short-term pregnancy outcomes through follow-up studies. Future research can address these limitations to improve and refine the risk prediction model, thereby enhancing its accuracy and practicality.

## 5 Conclusion

In this retrospective cohort study, age, body mass index, number of oocytes retrieved, EMT on HCG day, AMH, and FORT were identified as risk factors for non-pregnancy in patients undergoing ART treatment. The risk prediction nomogram model constructed based on these factors demonstrated predictive value for the risk of adverse pregnancy outcomes in patients, with an AUC of 0.911, which is competitive with existing models, and offers a clinically useful tool via a visual nomogram. Early clinical screening of high-risk populations using this integrated model holds significant value.

## Data Availability

The original contributions presented in the study are included in the article/supplementary material, further inquiries can be directed to the corresponding author.
